# How to Set Focal Categories for Brief Implicit Association Test? “Good” Is Good, “Bad” Is Not So Good

**DOI:** 10.3389/fpsyg.2016.00038

**Published:** 2016-02-03

**Authors:** Yuanyuan Shi, Huajian Cai, Yiqin Alicia Shen, Jing Yang

**Affiliations:** ^1^Key Laboratory of Behavioral Science, Institute of Psychology, Chinese Academy of SciencesBeijing, China; ^2^University of Chinese Academy of SciencesBeijing, China; ^3^Department of Psychology, University of WashingtonSeattle, WA, USA; ^4^College of Tourism, Huaqiao UniversityQuanzhou, China

**Keywords:** Brief Implicit Association Test (BIAT), Implicit Association Test (IAT), focal category, validity, implicit measurement

## Abstract

Three studies were conducted to examine the validity of the four versions of BIATs that are supposed to measure the same construct but differ in shared focal category. Study 1 investigated the criterion validity of four BIATs measuring attitudes toward flower versus insect. Study 2 examined the experimental sensitivity of four BIATs by considering attitudes toward induced ingroup versus outgroup. Study 3 examined the predictive power of the four BIATs by investigating attitudes toward the commercial beverages Coke versus Sprite. The findings suggested that for the two attributes “good” and “bad,” “good” rather than “bad” proved to be good as a shared focal category; for two targets, so long as they clearly differed in goodness or valence, the “good” rather than “bad” target emerged as good for a shared focal category. Beyond this case, either target worked well. These findings may facilitate the understanding of the BIAT and its future applications.

## Introduction

Researchers have longed for good measures of individual differences in implicit constructs. A good measure should not only be valid, but also be parsimonious. As a new variant of the Implicit Association Test (IAT, Greenwald et al., 1998), the Brief Implicit Association Test (BIAT, Sriram and Greenwald, 2009) possesses such qualities. The BIAT exhibits psychometric properties and usefulness comparable to the IAT (Sriram and Greenwald, 2009; Bar-Anan and Nosek, 2014; Yang et al., 2014). In contrast to the seven-block regular IAT, however, BIAT consists of only two combined blocks and in total uses fewer trials. Moreover, the BIAT uses a focal stimuli design, which allows participants focus on only two of the four categories but ignoring the remaining two categories. Such a design highlights a question of methodology: how to set the focal categories? Actually, this is the question we attempt to address in this research. In doing so, we focus on attitude BIAT because implicit attitude is the most investigated topic in implicit social cognition.

An attitude BIAT (e.g., flowers versus insect and good versus bad BIAT) usually includes two combined blocks: one compatible (e.g., flowers + good) and one incompatible (e.g., flowers + bad). Each of the two blocks involves four categories of stimuli, which fall into two contrasting pairs: one target pair (e.g., flowers versus insects) and one attribute pair (e.g., good versus bad). In each combined block, however, only one target and one attribute category are focal and need to be attended (e.g., flowers and good), with the remaining two categories as background (e.g., insects and bad). Thus, The focal stimuli involve three of the four categories: a pair of contrasting categories that serve as separate focal categories in two blocks, known as contrasting focal categories, and a third focal category from the other contrasting pair would be focal in both blocks, referred to as a shared focal category. In all, there are four versions of the BIAT: two versions use attribute categories (good or bad) as a shared focal category but target categories as contrasting focal categories, here called BIAT-Good (“good” as shared focal category) and BIAT-Bad (“bad” as shared focal category), and the other two versions using target categories (flowers or insects) as shared focal categories but attribute categories as contrasting focal categories, here called BIAT-Flower (“flower” as shared focal category) and BIAT-Insect (“insect” as shared focal category). The four BIATs involve the same four categories but differ in the pairings of categories that need to be attended to in each block (see **Table 1**).

**Table 1:** The designs of BIATs with different focal categories.

BIAT	Block 1	Block 2
**Flower versus insect BIAT (Study 1)**		
BIAT-Good	**Flowers + *good*,** Insects + bad	**Insects + *good***, Flowers + bad
BIAT-Bad	Flowers + good, **Insects + *bad***	Insects + good, **Flowers + *bad***
BIAT-Flowers	***Flowers* + good,** Insects + bad	Insects + good, ***Flowers* + bad**
BIAT-Insects	Flowers + good, ***Insects*** +** bad**	***Insects* + good**, Flowers + bad
**Ingroup versus outgroup BIAT (Study 2)**		
BIAT-Good	**Ingroup + *good***, Outgroup + bad	**Ingroup + *good***, Outgroup + bad
BIAT-Bad	Ingroup + good, **Outgroup + *bad***	Ingroup + good, **Outgroup + *bad***
BIAT-Ingroup	***Ingroup* + good,** Outgroup + bad	Ingroup + good, ***Outgroup* + bad**
BIAT-Outgroup	Ingroup + good, ***Outgroup* + bad**	***Ingroup* + good**, outgroup + bad
**Coke versus sprite BIAT (Study 3)**		
BIAT-Good	**Coke + *good*,** Sprite + bad	**Sprite + *good***, Coke + bad
BIAT-Bad	Coke + good, **Sprite + *bad***	Sprite + good, **Coke + *bad***
BIAT-Coke	***Coke*** +** good,** Sprite + bad	Sprite + good, ***Coke* + bad**
BIAT-Sprite	Coke + good, ***Sprite* + bad**	***Sprite* + good**, Coke + bad

*For each BIAT, bolded are focal categories; bolded and italic are shared focal categories*.

The purpose of our research is to examine whether the four versions of the BIATs are equally valid as measure of individual difference or whether the choice of focal category would influence the validity of the BIAT. We are not the first to address this issue. Besides providing initial evidence for the usefulness of the BIAT as a measure of attitude, identity and stereotype, Sriram and Greenwald (2009) also found that properties of the BIAT vary with the choice of focal categories. In their Experiment 1, they used BIAT to measure attitudes toward Kerry versus Bush as well as identification with male versus female, finding that for the attitude BIAT, “good” as a shared focal category outperforms “bad” (or BIAT-Good outperforms BIAT-Bad) and for the identity BIAT, “self” as a shared focal category outperforms “others” (or BIAT-Self outperforms BIAT-Other) in terms of reliability and implicit-explicit correlation. Sriram and Greenwald (2009) replicated these findings in Experiment 2. They also examined BIAT as a measure of various stereotypes in Experiments 3 and 4, but found that for the stereotype BIAT, using either attribute category (e.g., science or art) as a shared focal category showed comparable validity. This outcome suggests that when the perceived valence of two categories is not strikingly different, both attributes work well as a shared focal category. In another study that attempted to examine score transformation algorithms and other methodology issues related to the BIAT, the researchers replicated past findings that “good” as a shared focal category is superior to “bad” in a political attitude BIAT, by using a variety of criteria such as (a) sensitivity to known group differences; (b) internal consistency; (c) relations with other implicit measures; and (d) relations with parallel self-report measures (Nosek et al., 2014).

These two studies suggest that in choosing the shared focal category, a “good” or relatively good attribute is better than a “bad” or relatively bad attribute as indicated by the attitude BIAT or identity BIAT (Experiments 1 and 2, Sriram and Greenwald, 2009; Study 6, Nosek et al., 2014). When the valence, however, does not clearly differ between two attribute categories, both attribute categories perform equally, as indicated by the stereotype BIATs (Experiment 4, Sriram and Greenwald, 2009). Overall, the choice of a shared focal category follows a basic rule: if two attributes are distinguishable according to their valence or goodness, the “good” or relatively good one is superior to the “bad” or relatively bad one; when the two attributes are not clearly distinguishable by their valence or goodness, choosing either as the focal category is fine. Hereafter, we refer to this circumstance as the *good-focal* rule.

Extant studies, however, have only examined BIATs with attributes as shared focal categories, that is, BIAT-Good and BIAT-Bad, leaving BIATs with targets as shared focal categories unexamined. For the two BIATs with target as shared focal category, which approach is better? Furthermore, are they comparable to BIAT-Good and BIAT-Bad? In this study, we proposed that the revealed *good-focal* rule is also applicable to BIATs with a target as shared focal category. We hypothesized that when two targets clearly differed from each other in overall valence or goodness, the BIAT that used a relatively good target as a shared focal category would come out better. However, when the overall valence or goodness of target categories is not obvious, the two BIATs would function at a comparable level, paralleling the findings from Sriram & Greenwald’s Experiment 4. The main purpose of our research then is to test these hypotheses.

To achieve our goals, we conducted three studies. The first two studies, one correlational and one experimental, examined the four BIATs when the two targets clearly differed from each other in terms of overall goodness (Friese and Fiedler, 2010). The third study examined what would happen when the overall valence of the two targets did not clearly differ from the other. In Study 1, we examined implicit attitudes toward flower versus insect and tested the criterion validity of the four possible BIATs with the standard IAT as criterion. In Study 2, we examined the experimental validity of the four BIATs by testing their sensitivity to the experimentally induced ingroup favoritism over outgroup. In Study 3, we examined implicit attitudes toward the beverages Coke versus Sprite and tested the predictive validity of the four BIATs with real consumer decision making as the criterion. Overall across these three studies, we expected that BIAT-Good and BIAT-Bad would follow the established *good-focal* rule, that is, BIAT-Good would outperform BIAT-Bad. Specifically, for Study 1, since flower is naturally more pleasant than insect, we expected that “flowers” would serve better as the shared focal category than “insects,” where BIAT-Flower is better than BIAT-Insect. For Study 2, since people usually have a more positive attitude toward ingroup than outgroup, we expected that the induced “ingroup” would outperform “outgroup” in serving as the shared focal category, BIAT-Ingroup being more sensitive to induced ingroup manipulation than BIAT-Outgroup. In Study 3, given that *“*Coke*”* and *“*Sprite*”* are not clearly distinguishable in terms of overall valence or goodness, we expected both of them to function equally well as a shared focal category whereby BIAT-Coke and BIAT-Sprite would be equally predictive of purchase intention. In addition, we also expected that BIAT-Flower (Study 1) and BIAT-Ingroup (Study 2) would be comparable to BIAT-Good, while both BIAT-Coke and BIAT-Sprite would work similarly well as BIAT-Good (Study 3). In short, we aimed not only to replicate the previous *good-focal* rule derived from BIAT-Good and BIAT-Bad, but also to confirm that it could be generalized to BIATs with target as a shared focal category. The Ethics Committee of the Institute of Psychology, Chinese Academy of Sciences provided approval for all three studies. Additionally, in all three studies, written informed consents from all participants were obtained prior to commencing the experiments.

## Study 1 the Criterion Validity of Biat

In Study 1, we examined implicit attitudes toward flowers versus insects simultaneously with IAT and four versions of the BIAT. Both IAT and BIAT involve four categories (flowers versus insects and good versus bad) and are supposed to measure the same construct, that is, relative attitudes toward flowers versus insects. If a BIAT is valid, it should be significantly correlated with the IAT that aims to measure the same construct.

### Method

#### Participants

One hundred and five college students (35 males), ranging in age from 18 to 28 years (*M* = 20.18, *SD* = 1.34) participated in the study. Each was paid CNY10 for their participation.

#### Measures

The designs of the four versions of BIAT (BIAT-Good, BIAT-Bad, BIAT-Flower, and BIAT-Insect) are displayed in **Table 1**. These four BIATs actually involved four combined blocks, each including 40 trials. In terms of focal category in each block, the four blocks were: flowers + good, flowers + bad, insect + good, insect + bad. We used the standard 7-block IAT (target: flower versus insect, attribute: good versus bad) as criterion. All BIATs as well as the standard IAT were supposed to measure implicit attitude toward flowers versus insects.

#### Procedure

Participants completed all implicit measures separately in quiet, private cubicles. The order of standard IAT and BIATs was counterbalanced. For the four BIATs, the order of these four blocks was counterbalanced. Also, for both the IAT and BIATs, the order of compatible and incompatible blocks was counterbalanced.

### Results

Three participants were discarded: one due to his high error rates (36.9%) and the other two due to too many trials with latency below 400 ms (56.2 and 13.8%), leaving 102 participants for formal analysis (33 male). Following previous studies (Greenwald et al., 2003; Cai et al., 2004), we used the improved *D* score as the measure of the four BIATs and IAT. Since BIAT did not include any practice blocks, in calculating the *D* score, we discarded the first four warm-up trials for BIAT as recommended by recent studies (Sriram and Greenwald, 2009; Nosek et al., 2014) and the first two for IAT as well as those with latency greater than 10000 ms or smaller than 300 ms. The *D* score was obtained by dividing the mean latency difference between compatible and incompatible blocks by the pooled standard deviation, with higher score indicating more positive attitude toward flowers versus insects. To estimate the reliability, for each BIAT and IAT, we first obtained two *D* scores based on trials with even trial number and trials with odd trial number, respectively; then we calculated the correlation between them (*r*) and further the corrected split-half reliability (2*r*/(1+*r*)). The corrected split-half reliability coefficients, means and standard deviations are displayed in **Table 2**.

**Table 2:** Means, SDs, reliabilities, and correlations in Study 1 (*N* = 102).

Measure	Mean	*SD*	Correlations				
			1	2	3	4	5
1. BIAT-Good	0.88	0.32	0.68				
2. BIAT-Bad	0.64	0.39	0.14	0.48			
3. BIAT-Flowers	0.85	0.32	0.64^∗∗^	0.47^∗∗^	0.49		
4. BIAT-Insects	0.68	0.36	0.39^∗∗^	0.62^∗∗^	0.09	0.52	
5. IAT	0.94	0.35	0.28^∗∗^	0.15	0.30^∗∗^	0.18	0.71

*Numbers along diagonal are reliabilities; ^∗∗^*p* < 0.01*.

All BIATs’ effects as well as the IAT effect were significantly greater than zero, all *t*s > 16.50, all *p*s < 0.01, suggesting all four versions of BIAT as well as IAT were able to detect the known positive attitude toward flowers (versus insects), which is consistent with previous findings (e.g., Greenwald et al., 1998). Replicating a previous study (Sriram and Greenwald, 2009), BIAT-Good was significantly correlated with IAT, but BIAT-Bad was not, suggesting that when “good” or “bad” was set as shared focal category, “good” was good but “bad” was not so good. As expected, BIAT-Flower was significantly correlated with IAT, but BIAT-Insect was not, suggesting here when one of two target categories was set as shared category, the relatively good target was good while the relatively bad target was not so good. In addition, BIAT-Flower and BIAT-Good were highly correlated with each other and correlated with IAT to a similar extent, showing that when the *good-focal* rule was applicable, a “good” attribute and a good target made no difference as shared focal category.

In summary^1^, Study 1 replicated previous findings that BIAT with “good” as a shared focal category was valid while BIAT with “bad” as a shared focal category was not so valid. Extending previous findings, BIAT with a good target (in our case, flowers) as a shared focal category was better than BIAT with a bad target (in our case, insects) as a shared focal category. Beyond these cases, a good target or “good” attribute worked equally well in serving as a shared focal category.

## Study 2 the Sensitivity of BIAT to Experimental Manipulation

The findings from Study 1 are totally in line with our expectations. They, however, only offer correlational evidence. In Study 2, we conducted an experiment to test our hypotheses. In doing so, we examined implicit attitudes toward an induced ingroup versus outgroup. It is well established that the experimentally induced group leads to immediate favoritism toward the newly established ingroup over the outgroup, known as the minimal group effect (Tajfel, 1970; Tajfel et al., 1971). We employed a name-memorization procedure to induce ingroup membership, that is, allowing participants 45 seconds to memorize members’ names that belong to a novel group (Greenwald et al., 2002; Pinter and Greenwald, 2011). We used BIAT to measure implicit favoritism to the newly formed ingroup and tested sensitivity to the induced favoritism. The more sensitive about the induced favoritism, the larger the BIAT effect would be.

### Method

#### Participants

Ninety-nine college students (32 males) with an age range of 18–28 years (*M* = 21.90, *SD* = 2.31) participated in the experiment. Each was paid CNY20 for their participation.

#### Procedure

Participants were randomly assigned to one of the two experimental groups: a red group or a green group. All participants completed the tasks in private and quiet rooms. First, participants were told to imagine that some students had been classified in two groups—a red group and a green group based on their preferences for artistic style. Then they were asked to memorize five member names in either one of the two groups (red or green group) for 45 seconds to facilitate following tasks. The Chinese names in both the red group (*Chen Yang, Fan Yao, Huang Juan, Xu Fei, Guo Wei*) and green group (*Wu Bin, Ye Na, Yang Ning, Yu Ying, Hu Fang*) are common names in China. While participants were performing the memorization task, they viewed the member names on the computer. After finishing the memorizing task, all the participants completed a name-group association task just as Pinter and Greenwald (2011) did in their experiments. The categorization task includes two blocks (30 trials for each block) which were to help participants associate the names to the corresponding group. For the first block, color was used as a clue for participants to classify each presented name. That is, names in the red group were displayed in red while names in the green group were displayed in green. Participants were asked to classify all the names, and more importantly, to learn the association between the names and their groups. After finishing the first block, participants completed a second block without color clues to further familiarize themselves with the membership behind the names. After completing the name-association task, participants finished four attitude BIATs, which included four combined blocks in total. All BIATs measured implicit attitude toward the green versus red group. **Table 1** displays the design of the four BIATs. The order of the four combined blocks was counterbalanced.

### Results

Like before, we used the *D* score as index of BIAT. We cleaned the data and calculated the *D* score identically as we did in Study 1. A higher score represented a more favorable attitude toward the group of memorized names or ingroup. Thus, for those memorizing red group member names, a high score suggested more favorable attitude toward the red group over the green group, while the opposite was true for those memorizing green group member names. The descriptive statistics for all groups are presented in **Table 3**, which also includes the corrected split-half reliability for all BIATs. Effects of BIAT-Good and BIAT-Ingroup were significantly greater than zero regardless of the memorized group was red group or green group (all *t*s > 10.86, all *p*s < 0.01), suggesting that both BIAT-Good and BIAT-Ingroup were effective in picking up the induced ingroup favoritism. The effects of BIAT-Bad and BIAT-Outgroup, however, were significant when the memorized group was red group (*t*s > 3.98, *p*s < 0.01) but not when the memorized group was green group (*t*s < 1.92, *p*s > 0.05), suggesting BIAT-Bad and BIAT-Outgroup were not always effective in tapping the induced ingroup favoritism. Next, we tested our hypotheses with sensitivity to minimal group manipulation as the criterion. A larger BIAT effect or *D* score suggested higher sensitivity of the BIAT^2^.

**Table 3:** Means, SDs, and reliabilities in Study 2 (*N* = 99).

BIAT	Red group (*N* = 51)		Green group (*N* = 48)		Reliability
	Mean	*SD*	Mean	*SD*	
BIAT-Good	0.62^∗∗^	0.35	0.62^∗∗^	0.37	0.78
BIAT-Bad	0.25^∗∗^	0.45	0.10	0.49	0.61
BIAT-Ingroup	0.58^∗∗^	0.31	0.59^∗∗^	0.37	0.68
BIAT-Outgroup	0.32^∗∗^	0.39	0.13	0.47	0.68

*^∗∗^*p* < 0.01*

We began our testing by testing whether the “good focal” rule held true for BIAT-Good and BIAT-Bad. To do so, we conducted a mixed ANOVA on *D* score with a BIAT version as the within-subject variable and the group (red versus green) as the between-subject variable. As expected, BIAT-Good was more sensitive to experimental manipulation than BIAT-Bad, *F*(1,97) = 68.85, *p* < 0.01, $\eta_{p}^{2}$ = 0.42. The red group and green group did not differ from each other significantly, *F*(1,97) = 1.33, *p* = 0.25, $\eta_{p}^{2}$ = 0.01, suggesting induced implicit favoritism was independent of the color of the ingroup. The interaction was also not significant, *F*(1,97) = 1.96, *p* = 0.16, $\eta_{p}^{2}$ = 0.02, suggesting the superiority of BIAT-Good over BIAT-Bad did not vary with the group that was memorized. These findings suggested that BIAT-Good was better than BIAT-Bad in terms of sensitivity to experimental manipulation, replicating findings from Study 1 as well as previous findings (Sriram and Greenwald, 2009; Nosek et al., 2014).

Second, we examined whether the *good-focal* rule would hold true for BIAT-Ingroup and BIAT-Outgroup. We conducted a second mixed ANOVA with a BIAT version as the within-subject variable and the group (red versus green) as the between-subject variable. Again as expected, BIAT-Ingroup was more sensitive to experimental manipulation than BIAT-Outgroup, *F*(1,97) = 70.46, *p* < 0.01, $\eta_{p}^{2}$ = 0.42. The main effect of the group was not significant, *F*(1,97) = 1.95, *p* = 0.17, $\eta_{p}^{2}$ = 0.02, suggesting induced implicit favoritism did not depend on the specific group. Interaction was significant, *F*(1,97) = 5.44, *p* = 0.02, $\eta_{p}^{2}$ = 0.05, suggesting the extent of superiority of BIAT-Ingroup over BIAT-Outgroup varied by the group. Additional simple analyses showed that for the red group, the BIAT-Ingroup effect was significantly larger than the BIAT-Outgroup effect, *t*(50) = 4.45, *p <* 0.01, *d* = 0.73. This result also held true for the green group, *t*(47) = 7.31, *p <* 0.01, *d* = 1.07, which suggested that in spite of the extent of the superiority of BIAT-Ingroup over BIAT-Outgroup or that of ingroup favoritism over outgroup that varied with the group, the tendency remained similar across groups. In short, a “good” category is better than a “bad” category as a shared focal category.

Finally, we tested whether BIAT-Ingroup was comparable to BIAT-Good. We did a third similar mixed ANOVA. Neither the BIAT version nor the group produced significant effect, *F*(1,97) = 1.58, 0.01, *p* = 0.21, 0.95, $\eta_{p}^{2}$ = 0.02, 0.00, respectively. The interaction was not significant either, *F*(1,97) = 0.01, *p* = 0.94, $\eta_{p}^{2}$ = 0.00. These findings suggested that BIAT-Ingroup and BIAT-Good performed equally well.

In summary, we replicated all findings from Study 1: BIAT-Good was better than BIAT-Bad, BIAT-Ingroup was better than BIAT-Outgroup, but BIAT-Good was comparable to BIAT-Ingroup, providing experimental evidence for the *good-focal* principle.

## Study 3 the Predictive Power of BIATs

So far we have obtained both correlational and experimental evidence to support our hypotheses, as suggested by the *good-focal* principle: “good” is valid, “bad” is not so valid. But the *good-focal* principle has a premise in that there should be apparent difference in the overall perceived goodness or valence of the two targets. In both Studies 1 and 2, this premise is obviously satisfied: overall, flowers are more pleasant than insects and the ingroup is more pleasant than the outgroup. However, when the overall perceived desirability of two targets is not clearly differentiated in, for instance, attitudes toward Coke and Sprite, what would happen? We examined this circumstance in Study 3. In this case, we expected that using either target category as a shared focal category would produce an equally valid measurement. Confirming this expectation would provide validity evidence for the *good-focal* principle from a different aspect on the one hand, and on the other hand, clarify the practical issue of how to design BIAT in specific situations in which the targets or concepts do not markedly differ in valence. We approached this question by examining the predictive capability of BIATs that measured implicit attitude towards Coke versus Sprite in predicting consumption decisions.

### Method

#### Participants

One hundred and fifty three college students (57 males) ranging in age from 18 to 29 years (*M* = 21.98, *SD* = 2.00) participated in the study. Each was paid CNY15.

#### Procedure and Measures

**FIGURE 1:**
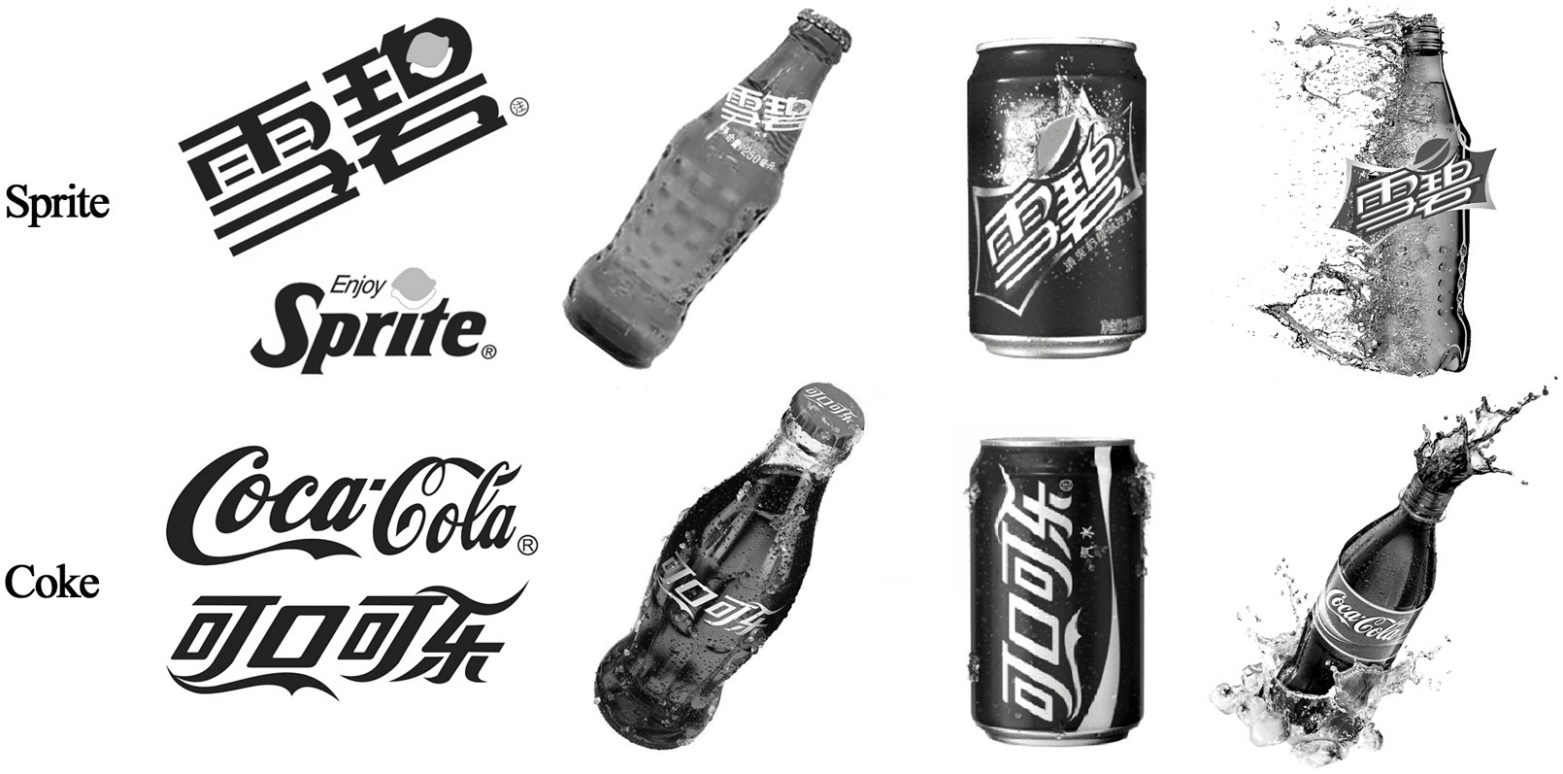
**Target exemplars in Study 3**.

The experiment was administered in a quiet and private room. First, all participants completed four BIATs to measure their implicit attitude toward Coke versus Sprite. The designs of the four BIATs are displayed in **Table 1**. In each BIAT, four images of each drink were used as category exemplars (see **Figure 1**). To minimize confusion arising from irrelevant factors, all pictures appeared in black and white. The order of the four combined tasks within BIATs was counterbalanced across subjects.

After completing the four BIATs, all participants were told that they would receive 5 bottles of the soft drinks as compensation for their participation. Six possible combinations existed for a grouping of 5 soft drinks: (1 = 5 *Sprites*, 2 = *4 Sprites + 1 Coke*; 3 = *3 Sprites + 2 Cokes*; 4 = *2 Sprites + 3 Cokes*; 5 = *1 Sprite + 4 Cokes*; 6 = *5 Cokes*). They were instructed to choose one combination from the six options. The consumption decision was used as the outcome variable in our analysis. Finally, they received the chosen drinks and were debriefed.

### Results

Again, we used the *D* measures for the four BIATs, with higher score indicating more favorable attitude toward Coke versus Sprite. The descriptive statistical information is displayed in **Table 4**. Among the four BIATs, two manifested positive effects, and two manifested negative ones. The average was 0.09 (*SD* = 0.24), which, although significantly different from zero, *t*(152) = 4.77, *p* < 0.01, was trivial because the *D* score is similar in meaning to Cohen’s *d* and a value below 0.15 is small (Cohen, 1988; Rudman, 2011). These findings were in line with our assumption that attitudes toward Coke and Sprite do not clearly differ from each other. In summary, people overall did not exhibit a clear preference for Coke or Sprite. Replicating previous findings, BIAT-Good predicted this consumer decision, but BIAT-Bad did not, suggesting when target categories were set as contrasting focal categories, “good” is valid as a focal category but “bad” is not so valid. However, when attribute categories were set as contrasting categories, both the BIAT with Sprite as a shared focal category and the one with Coke as a shared focal category were predictive of consumer decisions, and to similar extent. These results suggested that when two target categories did not differ significantly from each other in valence, either can serve as a shared focal category. Again, the predictive power of BIAT-Good, BIAT-Coke, and BIAT-Sprite proved to be comparable with the others.

**Table 4:** Means, SDs, reliabilities, and correlations in Study 3 (*N* = 153).

Measure	Mean	*SD*	Correlation			
			1	2	3	4
1. BIAT-Good	-0.07	0.42	0.67			
2. BIAT-Bad	-0.04	0.56	0.36^∗∗^	0.83		
3. BIAT-Coke	0.18	0.47	0.67^∗∗^	0.74^∗∗^	0.73	
4. BIAT-Sprite	0.29	0.45	0.65^∗∗^	0.75^∗∗^	0.49^∗∗^	0.68
5. Consumer decision	3.29	1.28	0.21^∗∗^	0.15	0.22^∗∗^	0.18^∗^

Numbers along the diagonal are reliabilities of the BIATs; *p < 0.05; ^∗∗^*p* < 0.01.

In summary^3^, we used BIAT to measure implicit attitudes toward Coke versus Sprite and took the consumer decision as the outcome. When the pair of targets was used as a contrasting focal category, we found results congruent with the *good-focal* rule. A novel finding is that when the perceived desirability of the two targets did not apparently differ from each other, either target was good as a shared focal category, whereby both BIAT-Coke and BIAT-Sprite worked similarly well.

## General Discussion

There are four variants of BIAT depending on how to set focal categories. In examining the properties of these four BIATs, we conducted three studies. In Study 1, we found that in measuring attitude toward flowers versus insects, BIAT-Good outperformed BIAT-Bad and BIAT-Flowers outperformed BIAT-Insects, whereas BIAT-Good and BIAT-Flowers functioned equally well. In Study 2, we found that in measuring attitude toward an experimentally induced ingroup, BIAT-Good was superior to BIAT-Bad and BIAT-Ingroup was superior to BIAT-Outgroup, and again, BIAT-Good and BIAT-Ingroup worked equally well. In Study 3, when measuring attitudes toward Sprite versus Coke, we found BIAT-Good to outperform BIAT-Bad while BIAT-Coke and BIAT-Sprite worked equally well since these beverages do not apparently differ in overall perceived goodness. These findings have important implications for the use and understanding of the BIAT.

Previous studies have suggested a *good-focal* rule exists for the BIAT when the shared focal category is one of the attributes. Across the three studies, we replicated this basic rule by examining diverse attitudes and using different criteria. We provided not only correlational evidence (Studies 1 and 3), but also, for the first time, experimental evidence (Study 2). Together with previous findings (Sriram and Greenwald, 2009; Nosek et al., 2014), our three studies provide further confidence that when attributes are used as a shared focal category, “good” should always be chosen, regardless as an outcome (Study 2) or as a predictor (Study 3).

Existing studies have exclusively employed BIATs that used attributes as a shared focal category, that is, BIAT-Good and BIAT-Bad. Our study assesses the validity of BIATs that also use targets as a shared focal category. Extending previous findings, we have obtained both correlational and experimental evidence that the revealed *good-focal* rule also holds true when using targets as a shared focal category. Specifically, when the two targets are clearly distinguishable in terms of overall goodness or valence, the relatively good target is valid whereas the relatively bad target is not so valid in serving as a shared focal category as indicated by the superiority of BIAT-Flowers over BIAT-Insects (Study 1) and BIAT-Ingroup over BIAT-Outgroup (Study 2). When the two targets are not clearly distinguishable, however, both targets function equally well as a shared focal category, as indicated by comparability of BIAT-Coke and BIAT-Sprite (Study 3). These findings provide a basic guide to design the BIAT when researchers want to use the attributes as contrasting focal categories, which is particularly helpful in some circumstances. For example, when one of the targets is vague in meaning, as in math versus other sciences, it might be better to use attributes as contrasting categories and the target of “math” as a shared focal category. People, however, may wonder how we could know which one of a pair of contrasting target is better. To determine this, we may first refer to results from past studies, just like the cases in our studies. If past studies do not provide a clear answer about whether one of the contrasting target is more pleasant than other one or not, a pilot study may be needed. If neither way produces a clear picture, both target categories may be equally suitable in serving as shared focal category in the BIAT.

Note that although the BIATs with “good” shared focal category in Studies 1 and 3 predicted criterion significantly (*r* = 0.28 and 0.30 for Study 1, *r* = 0.21 for Study 3) but the BIATs with “bad” shared focal category did not (*r* = 0.15 and 0.18 for Study 1, *r* = 0.15 for Study 3), all relationship differences were not significant for each corresponding comparison (*z* = 0.95, 0.88, 0.55, *p* = 0.34, 0.38, 0.58, respectively). These non-significant relationships might have compromised our conclusion. The pattern, however, is consistent across three examinations: “good” category outperform “bad” category in serving as shared focal category, which suggests that the non-significant differences may be due to insufficient statistic power. To examine if this is the case, we conducted a meta-analysis. The mean of three correlations between BIAT with “good” category as shared focal category and criteria is 0.26, with a 95% confidence of single-tailed lower bound being 0.19 (p. 74, Rosenthal and DiMatteo, 2001), which does not include the average of three correlations between BIAT with “bad” category as shared focal category and corresponding criteria (*r* = 0.16). This finding, together with the experimental evidence from Study 2, suggests that BIAT with “good” category as shared focal category is significantly better than those with “bad” category as shared focal category.

Moreover, we found that the BIAT with a good attribute as a shared focal category functions similarly to one with a relatively good target as a shared focal category. In Study 1, BIAT-Good and BIAT-Flowers are highly correlated with each other and similarly valid. In Study 2, BIAT-Coke and BIAT-Sprite are similarly sensitive to newly developed associations. In Study 3, BIAT-Good, BIAT-Coke and BIAT-Sprite are highly correlated with each other and similarly predictive of consumer decisions. These findings have important practical implications. To date, all existing applications of the BIAT exclusively have chosen shared focal categories from attributes. The explanation for this might rest on the fact that researchers are not sure if BIAT with a target as a shared focal category works and is comparable to BIAT with an attribute as a shared focal category. Our studies alleviate doubt by demonstrating that both designs are feasible and exchangeable. With these findings in mind, researchers have more choices accessible to them when designing their BIAT and can be more confident in using a target as a shared focal category in the future.

It is well known that the IAT is a relative rather than absolute measure of implicit attitude. Based on the structures of the two BIATs using a target as a shared focal category (e.g., BIAT-Flowers and BIAT-Insects), people may think that the two BIATs can assess absolute attitude toward the shared focal target (e.g., flowers or insects) along the evaluative continuum of good versus bad. Superficially, this sounds plausible. Empirical findings, however, suggest otherwise. In Study 1, BIAT-Flowers are significantly correlated with both BIAT-Good and the regular IAT, both of which are relative measures. Moreover, a regression with both BIAT-Good and BIAT-Flowers as predictors and the regular IAT as an outcome found that although the regression is significant [*R*^2^ = 0.10, *F*(2,99) = 5.55, *p* < 0.01], neither of the unique contributions of predictors is significant (both *t*s < 1.56, *p*s > 0.05), suggesting BIAT-Good and BIAT-Flowers are measuring identical constructs. In Study 3, BIAT-Coke and BIAT-Sprite are significantly correlated with each other as well as with both BIAT-Good and consumer decisions, both of which are again relative measures of the preference for Coke over Sprite. Further, in simultaneously predicting consumer decisions, although overall prediction is significant (*R*^2^ = 0.06, *F*(3,149) = 3.06, *p* = 0.03), none of the BIAT-Good, BIAT-Coke or BIAT-Sprite exhibits a significantly unique contribution (all *t*s < 1.25, all *p*s > 0.05), suggesting BIAT-Good, BIAT-Coke and BIAT-Sprite are measuring identical constructs. In summary, what BIAT measures is relative attitude toward the two contrasting targets rather than absolute attitude toward either of them.

As a kind of measure based on response latency, practice may influence IAT and BIAT effect (Nosek et al., 2014). In all three studies, we have participants completed at least four BIATs (four combined blocks in a sequence). People may think experiences obtained from the former blocks may help participants finish the latter ones more easily and ultimately contribute to the BIAT effects; that is, the order of BIATs may confound our results (Sriram et al., 2010). To address this possibility, we examined order effect in each of our studies. In Study 2, our analysis revealed no significant order effects. In Studies 1 and 3, we re-analyzed the relationship between each BIAT and corresponding criterion with BIAT order as a control variable and found all the main findings (as well as its patterns) remain similar, demonstrating the robustness of the *good-focal* rule.

People also may wonder what is the underlying mechanism of the *good-focal* rule, or the advantage of “good” over “bad” as a shared focal category. Basically, the four versions of the BIAT are almost the same: all involve the same four categories and map these categories onto the same computer key. The only difference is that participants are instructed to pay attention to different pairs of categories and to overlook other categories. Why do BIATs with different focal categories have different properties? One account derives from a prior work by Unkelbach et al. (2008). According to this work, positive information is more densely stored in the human memory than negative information and the associations between different positive information are stronger than those between different negative information. As a result, positive associations should not only be relatively separate from negative associations but also be more salient than negative ones (Ashby et al., 1999; Axt and Nosek, 2014). Thus, the association between attitude target and positive valence should be more prominent than the association between attitude target and negative valence. Ultimately, the differences in cognitive representation between positive and negative associations might have led to the superiority of “good” over “bad” as a shared focal category in the BIAT. However, empirical examination of this explanation is still lacking.

A primary limitation of our research is that in assessing the properties of the BIAT, we have only examined validity evidence of the BIAT, in particular, criterion validity in Study 1, experimental validity in Study 2 and predictive validity in Study 3. This research could benefit from using a more diverse set of criterions (see Sriram and Greenwald, 2009; Nosek et al., 2014). Validity, however, is the core property of a psychological measure. Hence, our demonstration of the *good-focal* rule in designing the BIAT still should be convincing. Together with previous studies (Sriram and Greenwald, 2009; Nosek et al., 2014; Yang et al., 2014), the usefulness of the *good-focal* rule in designing BIAT has been demonstrated for both target and attribute categories, in diverse domains of implicit social cognition such as implicit attitude, stereotype and identity and in both Eastern (e.g., China) and Western (e.g., America) cultures, suggesting the generalizability of the rule.

## Conclusion

The choice of focal category influences the properties of the BIAT. What matters above all is the choice of the shared focal category. In choosing a shared focal category, the *good-focal* rule should always be followed only if a “good” category exists. That is, if possible, one should always choose the “good” category as the shared focal category; when there is no obvious difference in perceived goodness or valence between potential candidates for shared focal categories, choosing either category, nevertheless, is fine.

## Author Contributions

HC conceived the idea; HC, YS, and JY designed the experiments; YS performed experiment 3 and JY performed Experiments 1 and 2; HC and YS analyzed the data and wrote the paper; YAS provided thoughtful inputs into the manuscript.

## Conflict of Interest Statement

The authors declare that the research was conducted in the absence of any commercial or financial relationships that could be construed as a potential conflict of interest.

## Footnotes

1 We also examined the semipartial correlations between BIATs and IAT controlling for two order variables: the order of IAT and BIAT, and the order of BIATs. Results showed that semipartial correlations of IAT with BIAT-Good (*sr* = 0.25, *p* < 0.01) and with BIAT-Flower (*sr* = 0.31, *p* < 0.01) were significant, but the semipartial correlations of IAT with BIAT-Bad (*sr* = 0.13, *p* = 0.20) and with BIAT-Insect (*sr* = 0.14, *p* = 0.16) were not significant, which were consistent with the findings based on zero-order correlations.

2 Before the formal significance tests, we examined whether the effect of each BIAT varied with its order. None of the order effects was significant, all *F*s(1,97) < 3.52, All *p*s > 0.05. So we did not include BIAT order as a variable in the formal analyses.

3 We also examined the semipartial correlations between BIATs and consumption decision controlling for the order of corresponding BIAT. Results showed that semipartial correlations of consumption decision with BIAT-Coke (*sr* = 0.29, *p* < 0.01), with BIAT-Good (*sr* = 0.20, *p* < 0.05) and with BIAT-Sprite (*sr* = 0.22, *p* < 0.01) were significant, but the semipartial correlation between BIAT-Bad and consumption decision was not significant, *sr* = 0.13, *p* = 0.12, which, again, were in line with the findings based on zero-order correlations.
